# “It’s good to feel like you’re doing something”: a qualitative study examining state health department employees’ views on why ineffective programs continue to be implemented in the USA

**DOI:** 10.1186/s43058-021-00252-4

**Published:** 2022-01-15

**Authors:** Stephanie Mazzucca, Louise Farah Saliba, Romario Smith, Emily Rodriguez Weno, Peg Allen, Margaret Padek, Ross C. Brownson

**Affiliations:** 1 Brown School, Washington University in St. Louis, St. Louis, MO 63130 USA; 2 Heluna Health, City of Industry, CA 91756 USA; 3 Bayer Strategy and Business Consulting, St. Louis, MO 63141 USA; 4 Department of Surgery, Division of Public Health Sciences, Alvin J. Siteman Cancer Center, Washington University School of Medicine, Washington University in St. Louis, St. Louis, MO 63110 USA

## Abstract

**Background:**

Mis-implementation, the inappropriate continuation of programs or policies that are not evidence-based or the inappropriate termination of evidence-based programs and policies, can lead to the inefficient use of scarce resources in public health agencies and decrease the ability of these agencies to deliver effective programs and improve population health. Little is known about why mis-implementation occurs, which is needed to understand how to address it. This study sought to understand the state health department practitioners’ perspectives about what makes programs ineffective and the reasons why ineffective programs continue.

**Methods:**

Eight state health departments (SHDs) were selected to participate in telephone-administered qualitative interviews about decision-making around ending or continuing programs. States were selected based on geographic representation and on their level of mis-implementation (low and high) categorized from our previous national survey. Forty-four SHD chronic disease staff participated in interviews, which were audio-recorded and transcribed verbatim. Transcripts were consensus coded, and themes were identified and summarized. This paper presents two sets of themes, related to (1) what makes a program ineffective and (2) why ineffective programs continue to be implemented according to SHD staff.

**Results:**

Participants considered programs ineffective if they were not evidence-based or if they did not fit well within the population; could not be implemented well due to program restraints or a lack of staff time and resources; did not reach those who could most benefit from the program; or did not show the expected program outcomes through evaluation. Practitioners described several reasons why ineffective programs continued to be implemented, including concerns about damaging the relationships with partner organizations, the presence of program champions, agency capacity, and funding restrictions.

**Conclusions:**

The continued implementation of ineffective programs occurs due to a number of interrelated organizational, relational, human resources, and economic factors. Efforts should focus on preventing mis-implementation since it limits public health agencies’ ability to conduct evidence-based public health, implement evidence-based programs effectively, and reduce the high burden of chronic diseases. The use of evidence-based decision-making in public health agencies and supporting adaptation of programs to improve their fit may prevent mis-implementation. Future work should identify effective strategies to reduce mis-implementation, which can optimize public health practice and improve population health.

**Supplementary Information:**

The online version contains supplementary material available at 10.1186/s43058-021-00252-4.

Contributions to the literature

- Mis-implementation, the continuation of ineffective public health programs or the premature termination of effective programs, can prevent public health agencies from achieving their mission of implementing programs to prevent and control chronic diseases. To understand how public health practitioners perceive ineffective programs and what they think contributes to the continuation of ineffective programs, we conducted in-depth interviews with 44 state health department practitioners working in chronic disease prevention and control.
- Practitioners characterized ineffective programs as those that were ill-suited for the target population, did not produce the intended change based on local evaluation data, lacked evaluation data, or were subject to staffing constraints that prevented implementation with fidelity. Many of the programs labeled ineffective by participants were those that have been found to be effective in research or other public health practice settings, which highlights the need for a robust understanding of the contextual factors required for a particular program to be successful in public health practice.
- Similar to previous research about program implementation and sustainability, factors related to a program itself, agency capacity for implementation, and funding availability were identified as important contributors to mis-implementation. Distinct from previous research, participants described the importance of factors including the effects of inertia, perceived sunk costs, and the desire to maintain strong partnerships, as well as the positive reinforcement practitioners receive for implementing a program, regardless of its effectiveness, in continuing the implementation of ineffective programs.
- This study contributes to both the understanding of why a program is ineffective and why an ineffective program is continued. Understanding public health practitioners’ views about the continuation of ineffective programs can inform future efforts to develop and test strategies to effectively prevent and reduce mis-implementation in public health practice.

## Introduction

Chronic diseases such as cardiovascular disease, cancer, and diabetes cause the majority of deaths worldwide and are costly to individuals, healthcare systems, and communities [1–3]. Governmental public health systems have been tasked with addressing the burden of chronic diseases by using evidence-based approaches to implement evidence-based programs and policies (EBPPs) that can improve modifiable chronic disease risk factors [4–7]. One approach is evidence-based public health, which is characterized by using the best available scientific evidence and information about the characteristics, needs, and preferences of the community to plan, implement, and evaluate programs and policies [8, 9]. Despite its known benefits, evidence-based public health is not used as often as it should be [9–11]—practitioners report that as many as 60% of programs implemented are not evidence-based [12, 13].

State health departments (SHDs) in the USA are one of three levels of governmental public health agencies in the USA—national, state, and local. The US Constitution grants much of the authority to protect the public’s health to the states. SHDs receive funds from national public health agencies, state-level legislatures, and other funders to implement chronic disease prevention and management programs and policies [14]. SHDs often act as the granting agency for local partners (e.g., local health departments, community-based organizations), which are responsible for delivering programs to individuals and communities. Decision-making in SHDs about implementing programs and policies is complex and varies by state but typically includes top leadership (e.g., department or division directors), middle managers, and programmatic staff. Within a SHD, chronic disease departments vary widely in terms of the level of hierarchy, specific positions and titles, and how governance is shared with local-level agencies [15]. Decision-making is likewise complex and shared among several types of practitioners: those in leadership positions (e.g., division directors, program managers) and those in lower-level positions (e.g., health educators).

Within SHDs, an emerging area of research is focused on mis-implementation, which contributes to our understanding of the underuse of evidence-based public health practices and strengthens efforts to promote its use [16]. Mis-implementation is defined as the inappropriate continuation of programs or policies that are not evidence-based or the inappropriate termination of evidence-based programs and policies [17]. It is important to study mis-implementation as a unique phenomenon since it may occur as a result of mechanisms beyond the absence of evidence-based public health, although both result in sub-optimal implementation of EBPPs. Cross-sectional studies have quantified the extent to which mis-implementation occurs in public health settings [17, 18] and identified practitioner- and organizational-level correlates of mis-implementation, such as individuals having the skills to modify programs or policies to a new population and the use of economic evaluation in decision-making about programs [18–20]. Additionally, a qualitative analysis of the same data presented in this paper identified key characteristics of agency leaders, e.g., being transparent and facilitating bidirectional communication, that can prevent mis-implementation [21]. These studies lay a strong foundation for the understanding of mis-implementation. To date, there has been little focus on understanding mis-implementation from the perspective of public health practitioners, including qualitative research to determine practitioners’ views of mis-implementation and why it occurs. Previous research focused on staff perspectives of program adoption, implementation, and sustainability has identified multiple factors influencing these outcomes, including the fit of a program with its target population, strong leadership and training opportunities, sufficient agency capacity and funding, and internal and external support for a program [22–25]. However, additional research is needed to understand practitioners’ views on what influences mis-implementation, which may be different than what supports program implementation.

Thus, the purpose of this study was to understand practitioners’ views about what makes a program ineffective and why ineffective programs sometimes continue to be implemented in public health practice. For this study, we focus on the inappropriate continuation of ineffective programs. Based on our previous work, the inappropriate termination of effective programs is primarily related to the lack of funding available for a program [17, 18], whereas there are many factors at multiple levels that influence the continued implementation of ineffective programs [18]. By focusing on practitioners’ perspectives on mis-implementation, this research can inform future work to develop and identify strategies to prevent or reduce mis-implementation that address the contextual factors that are key drivers of mis-implementation and that are relevant to practitioners.

## Methods

This qualitative study used a qualitative description approach to understand the perspectives of those with first-hand experiences with mis-implementation in governmental public health agencies [26]. A constructivist/interpretivist paradigm was used to guide the development of the interview guide questions, analysis, and interpretation because the research study was designed to understand and gain insights about how employees within governmental public health agencies, which are different across the USA, perceive and experience mis-implementation within their workplaces [27].

### Interview recruitment

For this study, states were purposively selected based on responses from a previously conducted quantitative survey that investigated implementation decisions in public health programs in all USA state chronic disease units of SHDs [18]. The states in this study (*n* = 8) were chosen to maximize variation, including states with lower and higher levels of self-reported frequency of mis-implementation, population size, and geographic representation from each of the four US Census Bureau regions (South, Midwest, Northeast, West). These contextual differences may influence public health practice; thus, it was important to obtain a variety of experiences to identify generalizable knowledge of mis-implementation. After selecting states, the research team contacted their chronic disease directors. The goal of this contact was to inform each director about the invitation that the research team would send to their employees. Directors were asked if they wanted their staff to participate and if they had any other contact suggestions besides those identified for the previously conducted national survey. If the director requested not to contact their employees, the research team replaced the state and repeated the approach.

All SHD chronic disease prevention or health promotion program staff in the eight sampled states, who participated in our national survey or were recommended by the chronic disease director, received an invitation to participate. The administrative staff were ineligible to participate, since they are typically not involved in programmatic decision-making. In the event that fewer than expected potential participants responded, the National Association of Chronic Disease Directors membership list was used to identify additional potential participants, and they were invited to participate. Participants were told that the purpose of the study was to learn about the factors that influence decision-making processes at SHDs to continue or end chronic disease programs. From the initial respondents, we asked for recommendations of additional contacts.

Interviews occurred between February 2019 and June 2019. Participants who completed the interview were offered a $40 Amazon gift card incentive or donation to a health-related non-profit organization from a list of options. This study was approved by the Washington University in St. Louis Institutional Review Board of the Human Research Protection Office (IRB# 201812062).

### Interview guide development

The interview guide questions focused on understanding the contextual factors associated with mis-implementation. The questions were developed based on the quantitative findings of the national survey previously conducted [16, 18]. These two data collection efforts, the national survey and interviews, were designed as part of a larger study to develop an agent-based model to understand mis-implementation from a systems perspective [16, 28]. The national survey used the social-ecological framework to understand potential factors related to mis-implementation. This framework, widely used in public health research, highlights the multi-level, biredictional influences on program implementation, whereby individual SHD staff characteristics, SHD-level factors, organizational capacity, and the external funding and policy environments all influence program implementation [29]. Interview guide questions were developed to build upon the survey findings and understand the influence of these factors in more depth. For example, interview questions asked why decisions were made within an SHD to continue an ineffective program and how key factors influenced the continuation of ineffective programs.

The interview guide questions were revised with input from the research team and stakeholder advisory board of public health practitioners. The development process included a pilot test with a member of our advisory board, a former SHD practitioner, to ensure the appropriateness of the length and language of the interview guide. The final interview guide consisted of open-ended questions about the perceptions of public health professionals working in SHDs’ chronic disease programs about the decision-making processes, reasons, facilitators, and barriers for continuing programs. Full text of the interview guide questions is available in Additional file 1. The interviews were conducted by trained research assistants over the telephone, and the interview guide was sent in advance to the interviewees. Each interview was audio recorded.

### Data analysis

Field notes were compiled after each interview to guide the interpretation of interview coding and to guide discussions to determine if thematic saturation had been reached. Each recorded interview was professionally transcribed using an online service (rev.com). Transcripts were de-identified by the research team and uploaded to NVivo (version 12).

We used a deductive approach for our thematic analysis [30]. An initial codebook was developed based on the interview guide questions. The initial codebook, particularly the initial set of sub-codes, was informed by the social-ecological framework used to develop the quantitative survey on which these interviews were based (Additional file 2). For example, in the parent code “Decision Making: why an ineffective program was continued,” sub-codes representing the multiple levels of influence on implementation were added to the initial codebook to facilitate deductive coding—i.e., program-level factors such as alternative programs available, agency-level factors such as capacity for implementation, community support, and political will/influence on a program. The codes and sub-codes of the first version were revised throughout the coding of the transcripts.

For the coding process, five research team members coded transcripts randomly assigned to them. All transcripts were submitted to consensus coding in pairs. Differences between coders were discussed and addressed. In case of lack of consensus, a third team member facilitated the process to achieve consensus. After consensus was reached between the pairs of coders on their assigned transcripts, the team members altogether identified and summarized sub-codes to investigate potential overlapping sub-codes and provided the needed adjustments. The final codebook consisted of nine codes and many sub-codes. While reviewing the codes and their coding reports, the saturation of the data was defined when all codes and sub-codes had a variety of data representing them, and few or no new concepts emerged from subsequent interviews. The presented work focuses on the themes from two parent codes: (1) why a program is considered ineffective and (2) what multi-level factors lead to its continuation.

## Results

A total of 44 interviews were conducted, with a range of 3 to 9 interviews per state. Interviews lasted between 20 and 68 min (average = 43 min). Nearly all (*n* = 43) interviewees were female. The average amount of time these practitioners had been in their agency and working in public health was 11 and 15 years, respectively. Most of the practitioners served as program managers or section directors, i.e., these interviewees were mainly middle managers. No discernable differences in themes were identified based on the reported level of mis-implementation or geographic region; thus, themes are presented for the overall sample.

The results are presented separately by major theme (i.e., code) and are summarized in Table 1. Themes are underlined in each section. First, we present practitioners’ conceptualizations of what makes a public health program ineffective, illustrating how practitioners define “ineffective.” Then, we summarize key reasons why practitioners believed ineffective programs continued to be implemented.

**Table 1:** Overview of themes

Theme	Theme description
*Major theme 1: Why a program is considered ineffective*	
Lack of program fit	Program was ill-suited to the populations served by the state health department.
No measured benefits	Local data demonstrated no or little positive outcomes, despite the program being evidence-based in other populations or settings.
Unknown effectiveness	Lack of evaluation data or the right type of evaluation data led to practitioner views that a program was ineffective.
Staffing constraints	Lack of sufficient staff or dedicated time to support successful program implementation or scale-up.
*Major theme 2: Why ineffective programs are continued*	
Inertia and sunk costs	Complex, grant-funded public health programs may be difficult to modify after implementation begins, and losing the money and time spent on initial implementation may decrease practitioners’ incentives to modify an ineffective program.
Information gaps	Agencies may not have adequate information to decide whether to continue a program, e.g., if evaluation was not built into the initial plan.
Desire to act	Public health staff may feel good that they are doing something to address a community concern, regardless of the effectiveness of the action.
Agency capacity	Agencies may lack the money, time, and personnel needed to identify, decide on, and implement changes to an existing program.
Program champions	The presence of someone in the SHD or community who is vocal about wanting a program to continue may challenge efforts to modify or discontinue an ineffective program.
Partnership maintenance	Ineffective programs may be continued to support strong relationships with community partners who are invested in a program.

### Why a program is considered ineffective

#### Lack of program fit

Practitioners described instances where a program was ill-suited to the populations they serve (i.e., a lack of external validity). As a result, the program, as implemented, does not reach the priority population who could benefit from it and/or does not improve the intended health outcomes. For example, the mode of delivery (e.g., in-person, group-based, or home visits) sometimes made participation in the program difficult for individuals, either because of logistical challenges of transportation or because of other challenges:

…so a lot of families are unwilling to do the program because they’re afraid of the city coming to their home, they’re afraid of landlord retaliation, so the numbers are pretty low. [Participant 1]

Several of the programs that were discussed as ineffective in this manner were those that have research evidence in support of their effectiveness and have been recognized nationally as an evidence-based program. For example, the Diabetes Prevention Program, which uses in-person, educational sessions and goal setting to decrease diabetes risk, was deemed ineffective because of the lack of implementation flexibility to better reach high-risk population groups who cannot attend multiple in-person sessions.

#### No measured benefits

Often, programs were described as ineffective because the local data did not show evidence of effectiveness, even though most were considered evidence-based programs, i.e., those that have a strong research base supporting their effectiveness or those that are included in the Community Guide. These programs were described as ineffective overall or as ineffective because specific components of the program did not achieve a given objective.

there hasn’t been really much change … in the past three surveys that were done. And they’re usually done approximately five years apart, so for a long time we haven’t really seen any major change at a population level... [Participant 5]

We’re hearing wonderful things from it but our numbers and the data that we’re seeing isn’t bearing it out. [Participant 6]

#### Unknown effectiveness

Some practitioners mentioned that there were programs that were deemed ineffective within their departments because there was a lack of evaluation data to measure and understand the impact of the program on individuals, even though the program may not have been truly ineffective:

I don’t think it was ineffective. We just couldn’t assess how much it was contributing… we couldn’t to the degree of saying, well, from $220,000 to $300,000 a year, we know that putting in that much amount was getting us this much impact. We couldn’t describe that adequately. [Participant 7]

…sometimes we get stuck in a rut when doing programs with same way, you know, year after year, and maybe not thinking about evaluating outcomes. ‘Cause it’s hard to evaluate [individual] outcomes from [an informational program]. [Participant 8]

One reason it’s ineffective is we cannot analyze and evaluate any kind of result ‘cause we just give it out. [Participant 9]

#### Staffing constraints

Practitioners noted that programs were often ineffective due to a lack of sufficient staff or dedicated time to support successful implementation or the scaling up of a program, throughout the processes of program planning and implementation. For example, there was not enough money to support necessary costs, such as ongoing support for those delivering programs, which resulted in the program being ineffective.

It just comes down to their capacity to actually do the necessary groundwork to make all those necessary connections. [Participant 2]

There has been a lot of movement on the federal level, so even our print materials aren’t necessarily up to date at this point. Just because there’s so much else going on, I don’t have a lot of time to focus on this project. [Participant 3]

Because we don’t have the capacity to go full blown, and really reach a significant number of people. [Participant 4]

### Why ineffective programs are continued

#### Inertia and sunk costs

Practitioners described the ideas of inertia and sunk costs when discussing why ineffective programs are continued. Practitioners highlighted that it was difficult to make changes to a program once it is already in place. As such, modifying an ineffective, already implemented program to improve its effectiveness would be too difficult. Also, if a program is funded and in place, many would be hesitant to end the program and lose the money and time already spent on implementing the program.

And you’re dictated by funding. It’s hard to change the course, especially three years into a five year grant. [Participant 17]

There’s a pretty hefty bureaucratic process to creating and maintaining contracts. So you had this ongoing thing that’s been approved time and time again. Each time you do it it’s just easier to get approved… And the way I kinda think about it is if you spend four months training for a marathon and then the week before you got shin splints, you might still run the marathon just because you put in all the work up to this point. [Participant 12]

#### Information gaps

The importance of program evaluation was discussed in relation to having adequate information to decide whether or not to continue a program. Some interviewees shared that there was not an evaluation built into the original implementation plan or there was one, but it did not capture the right set of outcomes at the right time, given the mechanism of change of the program. For example, only shorter-term outcomes were collected for a program that would have needed longer-term outcomes to demonstrate effectiveness. Also, there was a discussion of challenges on how program evaluation data could be used to inform decisions about whether to continue, modify, or discontinue a program. In one instance, there was an evaluation plan, but the data were reviewed too far into the grant period to make any changes to the program.

I think we just needed more time and more information from the field. [Participant 22]

I think for this was the struggle because we hadn’t put in an evaluator to the intervention from the start or the minimum an evaluation plan that would lead back to an evaluator at determined time. It was really hard for us. We didn’t have a baseline. [Participant 7]

#### Desire to act

Practitioners discussed the idea that it feels good to know that at least something is being done to address a public health concern, even if the program is not effective for its intended outcome and especially when there are no alternative programs that fit the disease, behavior, or population.

…at least we’re doing something or at least we’re out there, that kind of attitude. [Participant 15]

But it made people feel good that they were doing something for a vulnerable population. [Participant 11]

#### Agency capacity

Several practitioners discussed the impact of agency capacity, e.g., money, time, personnel, on decisions to continue ineffective programs. Some expressed concern that the decision to end a program, ineffective or effective, would put further strain on limited resources. For example, ending a program would force a workgroup to find other salary support for all employees funded by that program or risk losing their job.

I think another thing that it somewhat went into our decision making, but it probably is something we could have looked at more, is even our own staffing capacity and how we could better leverage other programs within the state because I think a lot of it, like [Respondent 1] mentioned, we just had so much staff turnover that we weren’t able to really dive in and make the changes in later parts of the grant. [Participant 19]

[The existing program] maintained a position and kept a certain level of funding coming in. [Participant 15]

I mean, I think a lot of the reason that it continued was just because that was a way to pay his salary. If it didn’t continue, then we were going to have to let him go because he couldn’t only work part-time through the other grant that he was through… I mean if we have somebody who’s willing to stay, we try to keep them as much as possible… [Participant 21]

#### Partnership maintenance

The main reason cited by practitioners for why an ineffective program was continued was that the continued implementation of a program, regardless of its evidence base, supported the maintenance of good relationships with community partners such as local health departments. Forcing community partners to make modifications to a program or ending a program could result in burning bridges with partners who were often the frontline implementers in communities.

Like they were excited about it, you know, like you don’t want to squash their excitement. [Participant 10]

…there can be incredible pushback to being told what to do, because that’s how people feel in the field. They feel like they’re being told what their community needs and what to do. [Participant 11]

…we don’t want to burn bridges and we want to continue to work with the people that we’ve been working with for a long time. [Participant 12]

#### Program champions

Additionally, practitioners noted that sometimes ineffective programs were continued due to the presence of program champions who advocated for the program. Program champions could be members of the community receiving the program, partnering organizations who implement the program locally, SHD staff, or policymakers who have influence over funding decisions.

It was hard to convince the commissioner at that time that we shouldn’t have it, … that this was not effective anymore and certainly is not sustainable because the health departments aren’t being paid for these visits. [Participant 13]

It’s a program that was, that had advocates from outside the department that established that even got the program going that convinced people that the program was needed and it was a program that was never going to accomplish it’s large stated goal. [Participant 11]

## Discussion

This study sought to understand SHD employees’ perspectives on why programs were ineffective and why some ineffective programs still continued to be implemented. To our knowledge, this is the first study to qualitatively explore why mis-implementation occurs from the perspective of public health practitioners. Themes discussed by practitioners aligned with the social-ecological framework used to guide the conceptualization of the study, in that practitioners described factors at multiple levels (i.e., program characteristics, staff-, agency-, and outer context-level factors) operating to influence mis-implementation. This is consistent with research identifying the multi-level determinants of low-value care, which is similar to the conceptualization of ineffective programs in this study [31]. Programs were typically ineffective if they were ill-suited to the population, could not be implemented sufficiently, or if they failed to reach those who could most benefit from the program. Some ineffective programs continued to be implemented because of internal decision-making dynamics, as a way to preserve relationships with partners, or because it was a way to keep staff fully funded.

This study extends the existing literature on mis-implementation, which has quantified the prevalence of mis-implementation in state and local health departments [17–20] and multi-level correlates of mis-implementation [18], and generated potential strategies from public health practitioners for ending ineffective programs [32]. Importantly, this study begins to fill an important gap in knowledge of public health practitioners’ perceptions of programs as something that should continue or end, as noted by Allen and colleagues [19]. These results provide novel information about public health practitioners conceptualize ineffective programs—in particular, programs that are not effective despite being evidence-based in other contexts. This indicates that additional work is needed to build robust evidence about effectiveness in diverse settings and the contextual factors that influence intervention effectiveness to aid public health practitioners in selecting an evidence-based program that will be effective in their community. Also, the qualitative findings in this study support the quantitative findings in Padek et al. of correlates of inappropriate continuation of ineffective programs [18]. Factors common between both studies include funding available and support from external audiences such as policymakers, program champions, and the general public. New in this study is the focus on maintaining strong partner relationships as a reason for continuing to implement ineffective programs and that practitioners are hesitant to jeopardize future collaborations, due to the importance of partnerships in public health practice [33]. Additional research is needed to determine how to address key contributors to mis-implementation, such as supporting public health practitioners in navigating the difficult conversations and negotiations with their partners about adapting or de-implementing ineffective programs.

These findings reiterate the importance of program-level factors (e.g., program fit, collecting evaluation data) that have been widely recognized in implementation science theories, including the Dynamic Sustainability Framework and the Consolidated Framework for Implementation Research [24, 34–37]. Of particular relevance to the findings in this study, the Dynamic Sustainability Framework “anchors the ultimate benefit of the intervention in terms of its ability to fit within a practice setting” and posits that the best fit of a program is achieved through ongoing evaluation of program effectiveness and contextual factors that inform refinements made to a program, ultimately leading to the sustainment of a program [34]. A greater focus on building skills of public health practitioners to develop, implement, and track effective refinements, i.e., adaptations, is needed [38], which can be facilitated by the longstanding attention in implementation science to adaptation [39, 40]. Adaptation in public health practice is complex and requires practitioners to have skills and resources to adapt evidence-based programs based on local evaluation data, and top leadership to buy into the value of adaptation, and funders to allow formal adaptations to be implemented. Often, state and local health departments in the USA are funded to deliver a particular program exactly as it was developed, with little flexibility for adapting programs. Recent efforts to support public health researchers and professionals as they plan for, implement, and track adaptations, such as the IM ADAPT tool (www.imadapt.org) and FRAME, have great potential to increase the use, tracking, and assessment of adaptation in public health practice [41, 42].

Practitioners described the influence of higher-level factors on mis-implementation, including agency capacity (e.g., dedicated staff), program champions, and maintaining relationships with external partners. These factors align with those that were previously identified as influential in the successful implementation of an evidence-based program [22, 23, 25, 43]. For example, a public health program sustainability framework developed by Schell and colleagues based on a review of empirical research and a concept mapping exercise identified several domains that correspond with the themes identified in this study, such as funding stability, partnerships, organizational capacity, program evaluation, and program adaptation [44]. These constructs were identified as contributing to the sustainability of evidence-based programs in public health practice; however, these same factors may also contribute to the sustainment of ineffective programs when incentives (e.g., funding) and infrastructures are in place for program implementation [31, 45]. Extending these previous studies, the emphasis on the emotional and relational influences on the continued implementation of an ineffective program beyond program and organizational factors identified in previous research is noteworthy. The typical focus of capacity-building efforts for public health practitioners is on individual skill-building and modifying organizational factors to support the successful implementation of EBPPs [46]. Specific to mis-implementation, it may be necessary to address additional concepts in these efforts, like the idea noted by participants that “it’s good to feel like you’re doing something” and how to effectively manage partnerships in instances where changes need to be made to a program about which partnering organizations or program champions feel strongly.

Themes from these interviews highlight how critical it is to prevent mis-implementation. Practitioners noted how difficult it was to make changes to a program or to discontinue it once it was implemented, discussed as the inertia effect. While evidence-based public health and mis-implementation are distinct concepts, the use of an evidence-based public health framework is a key strategy to prevent mis-implementation [8, 9]. The evidence-based public health approach includes the collection of local data to gain insights about the health problem within a community and the contextual drivers of the problem; matching this information with the best available evidence to prioritize program options, implementation of a program, and evaluation of the program [8]. Using an evidence-based public health approach may prevent some of the problems associated with mis-implementation discussed by these practitioners, such as the program not fitting with the context of their community, a lack of evaluation data to understand whether or not a program was effective, and the influence of stakeholders such as program champions and community partners on mis-implementation. Leaders within public health agencies play an important role in setting the expectations for using an evidence-based public health approach [19, 47–49]. Leadership support may be especially important in preventing mis-implementation, as the expectation and support for using ongoing, evidence-based decision-making could ameliorate the effects of inertia and sunk cost on decisions to continue implementation of ineffective programs. Previous research has focused on developing, testing, and disseminating strategies to increase the use of evidence-based public health [50–56], and future research and practice efforts should continue to determine the best ways to support the use of evidence-based public health in governmental public health agencies [57].

If mis-implementation cannot be prevented and programs cannot be adapted, lessons from the emerging area of de-implementation may be useful for reversing mis-implementation, by ending ineffective programs [57–60]. De-implementation has been primarily focused in clinical settings [59] on low-value and ineffective programs. Merging research about de-implementation in public health and social service settings can guide the de-implementation of ineffective programs by funders, SHDs, and contracted local implementing agencies [57]. McKay and colleagues suggest a stepwise fashion for de-implementation: identifying the programs that should be de-implemented, assessing the context for de-implementation, actively de-implementing, and evaluating the de-implementation process [57]. As in other aspects of implementation research, stakeholders’ buy-in for de-implementation will be critical [61] given that SHD practitioners were willing to allow ineffective programs to continue in part to maintain relationships with partner organizations and due to the presence of a program champion. Future work should focus on developing strategies to address the social and organizational contexts that facilitate de-implementation where mis-implementation is occurring.

Several limitations of this study should be taken into account when interpreting the findings, including generalizability due to selection bias, defining ineffective programs, and privacy concerns. States were selected based on responses to a national survey, which did not have equal response between states, and not all divisions within a SHD were interviewed. Thus, our results may not generalize to those states that were not selected or to the entire SHD. In addition, many of our respondents were middle managers, who are at the intersection of the day-to-day operations of a program and hold some decision-making power and are uniquely suited to answer the questions that relate to program mis-implementation and organizational-level factors influencing it [62]. However, the perspectives of those who are closer to the frontlines of program implementation, who were less represented in this study, may be different from those of middle managers; future research should seek to obtain their perspectives and understand differences by position. Also, we did not objectively assess whether or not the programs discussed were ineffective (i.e., that the program lacks research evidence supporting its effectiveness) and instead defined ineffective programs according to the perspectives of the interviewees. Last, although interviews were considered confidential, interviewees sometimes expressed hesitancy in divulging certain information that if shared, may have had funding or political consequences.

## Conclusion

The qualitative results presented in this paper contribute to and extend our understanding of mis-implementation in public health practice. A novel finding of this work is that SHD practitioners described ineffective programs as those that were ineffective within their local communities. As such, careful consideration should be given to what “evidence-based” means. The disconnect between what researchers and federal funders deem as an evidence-based program and what public health practitioners view as evidence-based within their communities could create tension in efforts to support the implementation of programs in public health practice. Additionally, practitioners in SHDs in the USA described multiple levels of factors that contribute to the continuation of ineffective programs—features of the program itself, agency capacity for implementation, relationships with partner organizations, and funding considerations. The description of these factors by practitioners highlights the importance of considering the emotional and relational implications of public health work.

The results from this study can inform the development of strategies to prevent or reduce mis-implementation in a manner that focuses on stakeholder-relevant contextual factors that contribute to mis-implementation. By identifying practitioners’ perceptions about mis-implementation and incorporating them into strategies to prevent mis-implementation and support the use of evidence-based public health, it is more likely that efforts will address salient contextual factors and be relevant to SHD practitioners. Future research is needed to identify effective strategies to address mis-implementation in public health practice and how to integrate them into governmental public health agencies, to optimize public health practice and ultimately improve population health.

## Supplementary Information

**Additional file 1.** Interview guide questions.

**Additional file 2.** Codebook.

## Data Availability

The datasets during and/or analyzed during the current study are available from the corresponding author on reasonable request.

## Abbreviations

CDC: Centers for Disease Control and Prevention
EBPPs: Evidence-based programs and policies
SHD: State health department
