# Supplementary material for: “It’s good to feel like you’re doing something”: a qualitative study examining state health department employees’ views on why ineffective programs continue to be implemented in the USA
Source: Implement Sci Commun. 2022 Jan 15;3:4. doi: 10.1186/s43058-021-00252-4 (PMC8760784; doi:10.1186/s43058-021-00252-4)
Supplement: Supplementary file 1 — Additional file 1. Interview guide questions. [file 43058_2021_252_MOESM1_ESM.docx]

**Additional File 1.** Interview Guide

**Background:** To start off, can you tell me a little bit about what you do at work?

**Probes:** What is your role within your work unit? Do you oversee programs? What types of programs do you oversee? What is your specific title and the name of your division/section/bureau? Do you supervise staff? If yes, roughly how many? How many people are in your unit?

- How long have you been in your current position?
- How long have you been with this agency or organization?
- How long have you worked in public health overall?

**Program Continuation**

1)  How would you define an ineffective program?

2)  Can you think of an example of an ineffective program in your state that continued that should have ended?

•  **If they answer “yes” to question 2, continue on to Option A.**

•  **If they answer “No” to question 2, skip to Option B.**

**Option A.**

Based on your response to the previous question, I am going to ask some more in-depth questions about that program and the decision-making process that occurred related to the [name of program].

3)  Can you briefly describe the ineffective program that should have ended and why it continued?

•  Why do you believe this particular program was ineffective?

•  What is the priority population? How long has it been going on in your state?

•  What do you wish would have happened with this program?

•  Is there something that could have been done that would have made the program effective?

4)  Was there a certain set of actions or decisions led to the continuation of the program?

•  Why was the choice made to continue [program] over something else or nothing?

•  What brings a program to a decision-makers attention? How frequently does this happen?

5)  Who (what roles of people) in your agency was involved in this decision-making process about the [insert name of program] program?

•  What was their position in your program or agency?

•  What was the role of leaders in your program or agency?

• Did leadership support you and others in the decision-making process?

6)  What sorts of factors played a role in the decision-making process?

•  Which factors were priorities? (If asked: Factors can be procedures for making decisions, influences, approval processes, etc.)

•  How were these factors identified?

•  What other factors should have been priorities?

7)  When deciding to continue [name of program], how did you learn about what worked in other areas (programming, population or geographical, or setting)?

•  Did you adapt this program?

•  How did you decide what was an appropriate adaptation?

8)  Thinking now about an ineffective program that your agency ended, what were the key reasons your agency decided to end the program? How is this different from the example we just discussed?

9)  Do you feel your agency is limited in their ability to use evidence-based interventions? What factors may be limiting your agency’s use of evidence-based interventions?

• What do you believe it would take your agency to be more evidence-based?

*For the purposes of this interview, we define evidence-based interventions as policies, environmental changes, system changes, programs, and services that have been evaluated and shown to work. These are policies, programs, and services that improve environments, systems, health behavior and health of communities and populations and improve health equity.*

• Who (what roles of people) would be the leaders involved with this change?

10) To what extent does the local/state/federal politics matter in this decision-making process? What is the influence of the county health officials, state legislature or governor’s office in your implementation of evidence based programs?

**[Go to Section C]**

**[Skip to this section if participant answered ‘No’ for Question 2].**

**Option B.**

Thank you. I am going to walk you through a hypothetical scenario in which your agency decides to continue an ineffective program. I am going to ask how you and your agency would go about making decisions on programs continuing or changes being made to programs. If at any time you feel uncomfortable answering the question, you can skip it.

**Scenario: *Say you have a physical activity promotion program that has been implemented statewide. This program has been in place for five years but recent data shows that the program has not made an impact in increasing physical activity nor physical activity knowledge with its intended audience. Your leadership has decided that they want to continue implementing this program despite this evidence.***

1) Who in your agency would be involved in the decision-making process around this program? • How frequently are these types of decisions-made

2)  What issues would bring a program to a decision-maker’s attention?

3)  What kind of factors would play a role in this decision-making process?

•  Probe as needed for decision-making procedures, approval processes, influences

•  How are these factors identified or applied in the decision-making process?

•  What are the priorities in decision-making processes?

•  What would the role of leadership be in finding an alternate approach?

•  How does leadership support you and others in program decision-making?

4)  When deciding to continue a program, how do you learn about what worked in other areas (programming, population or geographical, setting)?

•  Would you adapt this program?

•  How would you decide what was an appropriate adaptation?

5)  Thinking now about an ineffective program that your agency ended, what were the key reasons your agency decided to end the program? How is this different from the example we just discussed?

6)  Do you feel your agency is limited in their ability to use evidence-based interventions? What factors may be limiting your agency’s use of evidence-based interventions?

• What do you believe it would take your agency to be more evidence-based? Who would be the leaders involved with this change?

*For the purposes of this interview, we define evidence-based interventions as policies, environmental changes, system changes, programs, and services that have been evaluated and shown to work. These are policies, programs, and services that improve environments, systems, health behavior and health of communities and populations and health equity.*

7) To what extent does the local/state/federal politics matter in decision-making processes about programs continuing? What is the influence of the county health officials, state legislature or governor’s office in your implementation of evidence based programs?

**[Go to Section C]**

**Section C. Organizational & Agency Decision-making capacity**

Lastly, this section is going to ask a little more detail, generally, on how decision-making around program continuation happens in your work unit. If at any time you feel uncomfortable answering the question, you can skip it.

1)  Does your department implement program planning and quality improvement processes for the department itself? (For example: Balanced Scorecard, Baldrige Performance Excellence Criteria (or state version), LEAN, Plan-Do-Check-Act)

•  If Yes: Please describe the processes.

•  If No: Please tell me what processes you are aware of or those being used in other departments.

2)  How often does your unit consider significant and substantive changes to chronic disease programming so that they are using the best available evidence?

• Do you feel these changes in programming are managed effectively? Why or why not?

3) Who decides what programs typically get discontinued? Where are they in the hierarchy of your agency?

• If you were able to change one thing related to the organizational structure/ hierarchy of your department around decision-making and its support of an evidence-based processes, what would it be?

4) What advice do you have for others who want to end an ineffective program?
