# Supplementary material for: “It’s good to feel like you’re doing something”: a qualitative study examining state health department employees’ views on why ineffective programs continue to be implemented in the USA
Source: Implement Sci Commun. 2022 Jan 15;3:4. doi: 10.1186/s43058-021-00252-4 (PMC8760784; doi:10.1186/s43058-021-00252-4)
Supplement: Supplementary file 2 — Additional file 2. Codebook. [file 43058_2021_252_MOESM2_ESM.docx]

| **Node** | **Code Name** | **Description** | **Corresponding Question(s)** |
| --- | --- | --- | --- |
| 1.0 | Background of Interviewee | | |
| 1.1 | Description of Work Role and Unit | Overview of work at SHD | Can you tell me a little bit about what you do at work?  What is your role within your work unit?  What is your specific title and the name of your division/section/bureau?  Do you oversee programs? What types of programs do you oversee?  Do you supervise staff? If yes, roughly how many?  What is your specific title and the name of your division/section/bureau? |
| 1.2 | Time in position | Length of time in current position | How long have you been in your current position? |
| 1.3 | Time at agency | Length of time at SHD | How long have you been with this agency or organization? |
| 1.4 | Time in PH | Length of time working in public health | How long have you worked in public health overall?  **Include all description of their public health work. If including information about time in other state agencies, code to 1.1 but if describing other work context (i.e. university or local health dept settings), only code to 1.4 |
| 2.0 | Ineffective Programs that continued | | |
| 2.1 | Definition of ineffective | Interviewee definition of an ineffective program | How would you define an ineffective program? |
| 2.2 | Example of ineffective program (yes or no) | Interviewee knows of an ineffective program in their state. Code: Yes/No | Can you think of an example of an ineffective program in your state that continued that should have ended? Yes/No |
| *2.2.1* | Yes, used option A |  |  |
| *2.2.2* | No, used option B |  |  |
| 2.3 | Describe ineffective program | Interviewee description of ineffective program | Can you briefly describe the ineffective program that should have ended and why it continued? (Op A)  How long did the program continue? |
| 2.4 | Reason program ineffective | Why is the ineffective program considered ineffective | Why do you believe this particular program was ineffective? (Op A) |
| 2.5 | Could program become effective | Could something have been done to make the program effective  What the interviewee wishes would have happened to the program | Is there something that could have been done that would have made the program effective? (Op A)  What do you wish would have happened with this program? (Op A)  **This refers more to the hypothetical rather than what actually happened |
| 2.7 | Decision making process for adaptations | Was the ineffective program adapted | Did you adapt this program? (Op A)  How did you decide what was an appropriate adaptation? ( Op A)  Would you adapt this program? ( Op B)  How would you decide what was an appropriate adaptation? ( Op B) |
| 3.0 | Decision Making: Why Program was Continued | | |
| 3.1 | Actions to continue | Actions that led to continuing the ineffective program | Was there a certain set of actions or decisions led to the continuation of the program? (Op A) |
| **3.2** | Reasons why program was continued | Reason for the ineffective program continuing | Why was the choice made to continue [program] over something else or nothing? (Op A)  Why was the choice made to continue [program] over something else or nothing? (Op A) |
| *3.2.1* | Partnerships | Partnerships was the reason for the ineffective program continuing | ‘’ |
| *3.2.2* | Funding | Funding was the reason for the ineffective program continuing | ‘’ |
| *3.2.3* | Political will/influence on program | Political will/influence was the reason for the ineffective program continuing | ‘’ |
| *3.2.4* | Program had champion(s) | Program champion was the reason for the ineffective program continuing | ‘’  Note: Champions can be outside or inside the state health department |
| *3.2.5* | Data/Evaluation | Having or not having data or an evaluation was the reason for the ineffective program continuing | “ |
| *3.2.6* | No alternative program | There not being an alternative to the program was the reason for the ineffective program continuing | “ |
| *3.2.7* | Community Support | Community support for the program was the reason for the program continuing | “ |
| *3.2.8* | Program had been going on for a long time | The program had been going on for a long time was the reason for the program continuing | “ |
| *3.2.9* | Agency capacity | Agency capacity was a reason for continuing the ineffective program | **To parallel with 6.2.9, to keep reasons parallel for continuing (3.2) or ending (6.2) ineffective programs  **This can refer to capacity inside and outside the health department. |
| 4.0 | Decision Making: Factors and Priorities | | |
| 4.1 | Factors and Priorities | What factors in the decision making process are priorities | Which factors were priorities? (Op A)  **Factors can be broader than just those that are stated as priorities (sometimes asked factors in decision-making, later asked which are priorities) What are the priorities in decision-making processes? (Op B) |
| 4.2 | Learn about what worked | How does he interviewee learn about what works in other areas | When deciding to continue [name of program], how did you learn about what worked in other areas (programming, population or geographical, or setting)? ( Op A)  When deciding to continue a program, how do you learn about what worked in other areas (programming, population or geographical, setting)? (Op B) |
| 5.0 | Decision Making: Leadership/Decision Maker/Politics | | |
| 5.1 | Decision-maker’s attention | What brings programs to decision-maker’s attention | What issues bring a program to a decision-maker’s attention? (Op A)  What issues would bring a program to a decision-maker’s attention? (Op B) |
| 5.2 | Involved in decision-making | Who is involved in the decision making process about continuing or ending programs | Who (what roles of people) in your agency was involved in this decision-making process about the [insert name of program] program? (Op A)  Who (what roles of people) in your agency would be involved in this decision-making process about the [insert name of program] program? (Op B)  Who decides what programs typically get discontinued? Where are they in the hierarchy of your agency? |
| **5.3** | Leadership support | Does leadership support employees in decision to continue or end programs | Did leadership support you and others in the decision-making process? (Op A)  How does leadership support you and others in the decision-making process? (Op B) |
| *5.3.1* | Leadership is supportive | Leadership supports the decision-making process | ‘’ |
| *5.3.2* | Leadership is not supportive | Leadership does not support the decision-making process | ‘’ |
| *5.3.3* | Inconclusive leadership support | Leadership is not unsupportive or supportive or is both | “ |
| 5.4 | Leadership alternative approach | Only for option B: What is the responsibility of leadership to find an alternative approach to the ineffective program | What would the role of leadership be in finding an alternate approach? (Op B) |
| 5.5 | Political Influence to leadership | How do politics influence the decision-making process | To what extent does the local/state/federal politics matter in this decision-making process?  ** Code 5.5 when generally referring to political influence on the department. Code 3.2.3 when referring to the specific political influence on that program |
| 6.0 | Ending Ineffective Programs | | |
| 6.0.1 | Yes, Different example of ineffective program that ended |  | **Code 6.01-6.03 once at first mention when know ineffective program ending example is same or difference from example of ineffective program continuing |
| 6.0.2 | Yes, Same example of ineffective program that ended |  | When same example is used, no need to code 6.1, but do code reasons to end ineffective program |
| 6.0.3 | No, no example of program that ended |  |  |
| 6.1 | Ineffective program that was ended | Description of ineffective program that ended |  |
| **6.2** | Reasons why ineffective program was ended | Reason for the ineffective program ending | Thinking now about an ineffective program that your agency ended, what were the key reasons your agency decided to end the program? How is this different from the example we just discussed? |
| *6.2.1* | Partnerships | Partnerships was the reason for the ineffective program ending | ‘’ |
| *6.2.2* | Funding | Funding was the reason for the ineffective program ending | ‘’ |
| *6.2.3* | Political will/influence on program | Political will/influence was the reason for the ineffective program ending | ‘’ |
| *6.2.4* | Program had champion(s) | Program champion was the reason for the ineffective program ending | ‘’ |
| *6.2.5* | Data/Evaluation | Having or not having data or an evaluation was the reason for the ineffective program ending | “ |
| *6.2.6* | No alternative program | There not being an alternative to the program was the reason for the ineffective program ending | “ |
| *6.2.7* | Community Support | Community support for the program was the reason for the program ending | “ |
| *6.2.8* | Program had been going on for a long time | The program had been going on for a long time was the reason for the program ending | “ |
| *6.2.9* | Agency Capacity | The agency did not have the capacity to carry out the program was the reason for the program ending. | “ |
| 6.3 | Advice | How interviewee recommends others to best end ineffective programs | What advice do you have for others who want to end an ineffective program? |
| 7.0 | Evidence-based Organizations | | |
| 7.1 | EBI use limited | How much the interviewee feels their agency is limited in its ability to use EBIs | Do you feel your agency is limited in their ability to use evidence-based interventions? |
| *7.1.1* | Yes, EBI use is limited | Yes, something is limiting EBI use | “ |
| *7.1.2* | No, EBI use is not limited | No, nothing is limiting EBI use | “ |
| *7.1.3* | Inconclusive EBI limitations | It is unclear if something is limiting EBI use or both | “ |
| 7.2 | EBI Use factors | Factors that enhance or limit EBI use at agency | What factors may be limiting your agency’s use of evidence-based interventions?  What do you believe it would take your agency to be more evidence-based? |
| *7.2.1* | Limiting factors | Factors that are limiting EBI use | “ |
| *7.2.2* | Needed to increase EBIs | What needs to be done to increase EBI use | “ |
| 7.3 | Leadership role in being evidence-based | What leaders would be involved in making the agency more evidence-based | Who (what roles of people) would be the leaders involved with this change? |
| 7.4 | Frequency considering change | How often does agency consider change to CD programming to use the best available evidence | How often does your unit consider significant and substantive changes to chronic disease programming so that they are using the best available evidence? |
| 7.5 | Changes managed effectively | Are changes to make programming more evidence-based managed well | Do you feel these changes in programming are managed effectively? Why or why not? |
| 8.0 | Organizational Factors | | |
| 8.1 | Quality Improvement | Does the agency use program planning and/or quality improvement processes | Does your department implement program planning and quality improvement processes for the department itself? |
| 8.2 | Change organizational structure | Change interviewee would make to the agency’s organizational structure around decision-making and supporting EBPs | If you were able to change one thing related to the organizational structure/ hierarchy of your department around decision-making and its support of an evidence-based processes, what would it be? |
| 9.0 | Good Quotes and Key Examples | Place any text that exemplifies a theme or a child node especially well. Will be used in later theme reports. | 9.0 always needs the topic/content coded as well to one of the codes above in sections 2 through 8  All 9.0 code should be double coded- the double code should reach consensus. |
